# You Don’t Bend It Like Beckham if You’re Female and Reminded of It: Stereotype Threat Among Female Football Players

**DOI:** 10.3389/fpsyg.2019.01963

**Published:** 2019-08-28

**Authors:** Hilmar Grabow, Melanie Kühl

**Affiliations:** Social and Political Psychology, Institute of Psychology, Kiel University, Kiel, Germany

**Keywords:** stereotype threat, football, soccer, motor task, precision, sport

## Abstract

Originally, the stereotype threat effect – poorer performance due to a fear of fulfilling a negative stereotype about one’s group – was demonstrated for cognitive tasks (e.g. Steele and Aronson, 1995, or Steele, 1997). Drawing on the widespread stereotype of women being unable to play football we experimentally tested (*N* = 80) whether a respective threat affected female football players’ goal scoring precision, i.e. a complex and demanding motor task. Those participants who were reminded of the stereotype scored significantly less hits than those not reminded. Additionally, deviations from the instruction during task execution (e.g. shooting from another distance than demanded or using the wrong foot) were recorded. Stereotype threat did not affect this comparatively more cognitive task of following instructions correctly. In order to explore underlying mechanisms of the observed stereotype effect, several potential mediators, e.g. measures of cognitive interference, or collective identification, were tested. None emerged as an unquestionable link between threat and motor performance. We discuss, however, why collective identification – in comparison to cognitive demand – appears to be the more promising explanatory concept.

## Introduction

Stereotypically, football^1^, the most popular sport worldwide (e.g. Brüggemeier, 2006, or Kunz, 2007), is perceived as male. According to Csizma et al. (1988), it ranks amongst the masculine – as opposed to neutral (e.g. tennis) or feminine (e.g. figure skating) – sports. The comment of Pfister (2015, p. 565) that ‘“Real” football is still men’s football’, concisely describes the status of women’s football in the media and public. Even at highest administrational level, women’s football is not taken seriously, as Sepp Blatter’s suggestions concerning an increase of its popularity illustrate. During his reign as FIFA president, he proposed – as reported by Christenson and Kelso (2004): ‘Let the women play in more feminine clothes like they do in volleyball’, explicating ‘They could, for example, have tighter shorts’. Historically, women were kept from playing football over considerable periods of time. In Germany, for example, the football association Deutscher Fußball-Bund (DFB) even banned it between 1955 (Hoffmann and Nendza, 2007b) and 1970 (Hoffmann and Nendza, 2007a). Women were judged unfit for this sport. This categorical verdict may have changed over time. In 2017, 5,819 women’s and 5,875 girls’ teams were active under DFB auspices, with over one million female DFB members (Deutscher Fußball-Bund, n. d.a). Women’s football, unfortunately, is still not taken seriously in relation to the men’s game, with it on many occasions being impeded, depreciated, and ridiculed (e.g. Keller, 2018, or Schwaiger, 2011), and viewed as ‘inappropriate for girls’ (Scraton et al., 1999). Football pundit Max Merkel did not, in all likelihood, utter an exclusive opinion but echo the mood of at least a considerable part of the football-oriented public, when he bad-mouthed women’s football as a ‘distress for the eyes’ (Merkel, 2003). Summing up, in Germany (and other countries), stereotypical ascriptions have developed over time. In the past, it was an established idea that women must not play football; nowadays, a widely shared assessment is that they are not (as) good (as men) at it.

Considering stereotypical characteristics of men and women, this predominant perception of football as male can hardly surprise: across many cultures, typically female characteristics comprise ‘timid’, ‘mild’, ‘soft-hearted’, ‘submissive’, or ‘fearful’ (Williams and Best, 1990). These appear to be incompatible with a physical competition like football. Typical male characteristics, however, are perfectly in line with it: ‘active’, ‘ambitious’, ‘strong’, ‘robust’, ‘quick’, or ‘tough’ (Williams and Best, 1990). Diekman and Eagly (2000) argue that gender-related stereotypes change over time, following perceived changes in social roles. For the present, however, they report that males are ascribed masculine personality (e.g. competitive, daring, aggressive, or courageous) and physical attributes (e.g. rugged, muscular, or physically strong) to a higher degree than females. Again, these ‘male’ attributes are perfectly in line with the requirements of football. Lueptow et al. (1995) report similar results: their participants on average rated being athletic as well as competitive clearly more typical male than typical female. In contrast to Diekman and Eagly (2000), Lueptow et al. (1995) consider gender stereotypes rather stable over time. According to Fiske et al. (2002), women are perceived to be warmer/more communal but less competent/agentic than men, i.e. viewed in the light of a paternalistic stereotype. Women are likely to suffer from a backlash effect if they do not fit into this stereotype (e.g. Rudman, 1998, or Rudman and Glick, 2001), which is highly likely for female football players who strive to excel in a typical male domain.

Football, indeed, requires athletic skills from the players. Therefore, the stereotype (the widely shared belief of women being bad at football) could be based on a better athletic performance of males in comparison to females (Knisel et al., 2009), mirroring average morphological and physiological differences between genders (Hottenrott and Neumann, 2012) such as muscle size (Högler et al., 2008). It is not reasonable to assume, however, that these differences directly impact other football-related competences like technical or tactical skills.

For the purposes of our study, the genesis of this specific negative stereotype is far less important than its potential effect on women’s performance. Generally speaking, ‘Stereotype threat is being at risk of confirming, as self-characteristic, a negative stereotype about one’s group’ (Steele and Aronson, 1995, p. 797). Traditionally, the stereotype threat literature has dealt with cognitive tasks (e.g. Steele, 1997, Aronson et al., 1999, Spencer et al., 1999, Cadinu et al., 2003). For a start, Steele and Aronson (1995) reported worse results in a verbal test for African Americans compared to Whites if the racial stereotype about intellectual ability was salient. Since then, the stereotype threat effect was demonstrated across many samples comprising members of diverse (social) groups, e.g. women compared to men (Spencer et al., 1999), African vs. White Americans (Steele and Aronson, 1995), or White vs. Asian men (Aronson et al., 1999). Tempel and Neumann (2016) provide evidence for a moderation of stereotype activation by gender role orientation: the more feminine their participants rated themselves the less well they performed on mental rotation or mathematical tasks if gender stereotypes were activated. Thus, stereotype threat effects appear to be more severe the more relevant (in terms of self-concept) the respective identity is. Furthermore, the effect is not restricted to purely intellectual tasks; Stone et al. found it for motor tasks in golf amongst Black and White participants and stated that ‘Blacks suffer when the stereotype concerning their supposed poor sports intelligence is made salient, whereas Whites suffer when the stereotype concerning their supposed natural athletic ability is made salient’ (Stone et al., 1999, pp. 1225–1226). Based on the premise that ‘Sport and athletics may represent one of the few domains in which Whites are stereotyped negatively and suffer psychologically as a result’ (Stone et al., 1999, p. 1213), they chose a miniature golf task in order to test whether different stereotypes would deteriorate the performance of both members of minorities as well as majorities. Indeed, the participants’ performance, measured by the number of strokes conducted in order to hit balls in a series of holes, suffered when participants were threatened by a fitting stereotype about their group. Not least because the participants were relative golf novices, the motor task in all likelihood was executed under articulate conscious control. In comparison to well-learned, automated movements, such explicit cognitive effort could be quite prone to cognitive interference – much like the cognitive tasks examined earlier in the stereotype threat literature. Chalabaev et al. (2008) avoided this potential problem by examining a dribbling task amongst experienced female football players, hence a well-learned routinely executed set of movements. The time for completion of a slalom course served as the dependent variable. Thus, results were not solely attributable to technical but also to athletic skills, hence, a dimension reflecting actual gender differences.

Summarising research findings, Keller (2008) extracted four preconditions whose presence increase the likelihood of a manifestation of the stereotype threat effect. First, a relevant stereotype has to be salient and directly applicable to the situation. Second, the respective person needs to highly identify with the domain (here: football). Third, the task has to be demanding, touching the person’s performance limit. Fourth, the person needs to have the impression their performance is under scrutiny, i.e. the test has to be diagnostic.

In line with this research, we predicted and found that, compared to an unthreatened control group, women kick the ball less precisely if reminded of their gender. We chose this specific dependent variable because it is not plausible to expect an actual gender specific difference in shooting precision (or technical skills in general). Thus, the threat could not plausibly initiate remembrance of factual knowledge about women’s power and strength – unlike, e.g. in the study by Chalabaev et al. (2008).

In addition to a conceptual replication of the already well-established stereotype threat effect (employing a new dependent variable), we tested potential explanations for its occurrence. Apparently, there has not yet emerged an undisputed model of the mechanisms behind it. Several concepts/variables have been tested as mediators between stereotype threat and decreased performance, e.g. anxiety, self-efficacy, or evaluation apprehension (Spencer et al., 1999), or, for sensorimotor tasks, monitoring processes (Schmader et al., 2008). The last-mentioned authors argue that a deterioration of automated sensorimotor tasks can hardly be accounted for by working memory impairments – according to their integrated process model an important link between stereotype threat and worsening performance on cognitive tasks under controlled processing. Therefore, they propose that under threat, monitoring and controlling of a person’s behaviour consume resources and impede performance (pp. 337–338).

In order to explain the expected effect of stereotype threat we draw on the social identity tradition, i.e. social identity theory (SIT, Tajfel and Turner, 1986), and self-categorisation theory (SCT, Turner et al., 1987). Tajfel and Turner present the concept of social categorisations – ‘cognitive tools that segment, classify, and order the social environment’ (Tajfel and Turner, 1986, p. 15), whereby individuals distinguish themselves (and others within the same category) from members of outgroups. Groups ensuing from these categorisations ‘provide their members with an identification of themselves in social terms’ (Tajfel and Turner, 1986, p. 16). Tajfel and Turner further assume that ‘Social groups or categories and the membership of them are associated with positive or negative value connotations. Hence, social identity may be positive or negative according to the evaluations’ (Tajfel and Turner, 1986, p. 16). Given that ‘Individuals strive to maintain or enhance their self-esteem: they strive for a positive self-concept’ (Tajfel and Turner, 1986, p. 16), being reminded of a decidedly negative evaluation of one’s group needs to be considered a setback for this striving. Potential ways out of this aversive state of a threatened social identity are individual mobility (leaving or dissociating oneself from the respective group), social creativity (e.g. referring to a different dimension when comparing in- and outgroup), or social competition, i.e. direct competition with the outgroup (Tajfel and Turner, 1986). According to Turner et al. (1987), individuals feature a bundle of cognitive representations of the self which ‘take the form, amongst others, of self-categorisations’ (Turner et al., 1987, p. 44); the salience of a specific self-concept is dependent on ‘an interaction between the “relative accessibility” of that categorisation (…) and the “fit” between the stimulus input and category specifications’ (Turner et al., 1987, p. 54).

Concerning the participants and the setting of our study, both relative accessibility and fit for a self-categorisation as female football players were highly likely. Being reminded of the negative stereotype should constitute a threat to this collective identity and activate strategies in order to maintain the individual participants’ self-esteem – e.g. dissociation from the group of female football players.

This reasoning led us to considering collective identification as a potential mediator of the hypothesised effect of stereotype threat on the motor task. In our analyses, we further examined the following potential mediators: cognitive interference, subjective vulnerability for sexist discrimination, and subjective task difficulty.

## Materials and Methods

### Procedure

**Figure 1:**
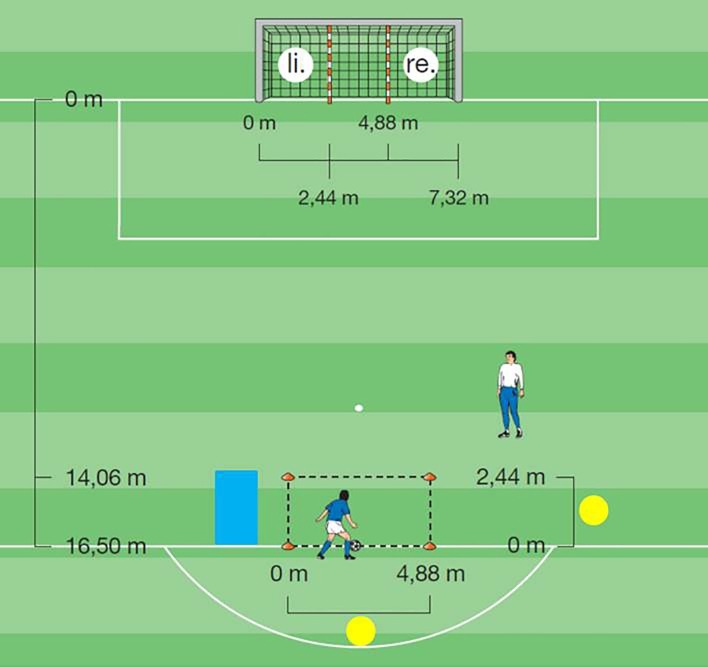
Test setting [picture taken from Deutscher Fußball-Bund (n. d.b), and slightly amended].

In order to recruit participants, managers of amateur women’s football teams in northern Germany were approached. Data collection took place between 15 January and 8 April 2016 at times of regular training sessions of the co-operating clubs. After preparation of the test setting (see Figure 1; the blue square indicates the placement of footballs at the beginning of each session, the yellow circles represent the position of the video cameras used to film the test), the experimenter and her assistant – both female – welcomed the participants. In order to standardise the setting as far as possible, the team managers (all of them male) were excluded from the test situation. Thus, no males were immediately present who could involuntarily trigger the social category ‘gender’.

Participants took pens and clip-boards containing background information about the study, a consent form, and a questionnaire from a basket. In particular, they were informed about the test session being video-recorded (in order to ensure the impression of the test being diagnostic). From the outside, the clip-boards were undistinguishable, thus ensuring a random self-allocation to experimental conditions.

The first part of the questionnaire comprised questions referring to age, footballing experience, self-efficacy, and subjective importance of the sport. The following instruction contained the experimental manipulation which was imbedded into a bogus description of the study’s scientific basis. The threatened participants read:^2^

*Although men and women do not directly compete playing football*, one can state on a scientific basis that *men outperform women in motor tasks* concerning force and velocity (Knisel, Opitz, Wossmann, and Ketelhut, 2009). *Research supposes that there are hardly differences between men and women concerning the capability characteristics concentration, aplomb, and precision.* During this study, the shooting precision *of women* shall be video-recorded and analysed in order to advance research.

The text for non-threatened participants differed:

*In the realm of football* one can state on a scientific basis that *there are individual differences in motor performance* concerning force and velocity (Knisel, Opitz, Wossmann, and Ketelhut, 2009). *In how far there are individual differences concerning the capability characteristic shooting precision has not yet sufficiently been researched*. During this study, the shooting precision shall be video-recorded and analysed in order to advance research.

Hence, stereotype threat (the sole independent variable: threat vs. no threat) was manipulated between participants. The rest of the study was exactly the same for both threatened and non-threatened participants. The manipulation was followed by a written instruction for the actual motor task (taken from Deutscher Fußball-Bund, n. d.b): kicking the ball with maximum force from a designated square into either the left or right target field of the goal (see Figure 1). More specifically, the participants were supposed to take a ball from a pile, place it in front of the designated square, drive it into the square (first touch), and kick the rolling ball with either their left or right foot into a pre-specified area of the goal – either the left- or right-sided target field (second touch). Before each trial, the experimenter told the participants which foot to use and which target field to aim for. In order to enable familiarisation with their task, participants executed one test run followed by eight experimental trials.

After completion of the motor task, participants filled in the second part of the questionnaire, consisting of items concerning the manipulation check, subjective vulnerability for sexist discrimination, collective identification, cognitive interference, and subjective difficulty of the task. Upon completion, participants were thanked and debriefed.

### Measures

#### Dependent Variables

The participants’ task was to kick a football into specified areas of the goal. They had eight tries, each. Thus, the first DV, the number of hits, could range between 0 and 8. Additionally, the experimenter assessed the correct (or incorrect) execution of the task per try. If the player used the incorrect leg for kicking, and/or shot from outside the designated square the try was noted as a mistake. Thus, the second DV, the number of mistakes, could also range from 0 to 8.

#### Additional Variables

Some data were collected before the experimental manipulation. Concerning demographics only age was measured. As a potential control variable, participants indicated the number of years of experience as active football players. Moreover, we asked them to rate the importance they assign to football (‘Football is very important to me.’ and ‘My sporting performance within the field of football is very important to me.’; (1) ‘does not apply’; (2) ‘does rather not apply’; (3) ‘rather does apply’; (4) ‘does apply’; Cronbach’s *α* = 0.788), and task-related self-efficacy (‘I always know what to do when the task is scoring a goal’, ‘I always achieve the task of scoring a goal when I try to.’, ‘I cope well with surprising goal-scoring tasks.’, ‘I am looking forward to goal-scoring tasks calmly because I can trust my abilities.’, ‘I never know how to deal with new goal-scoring tasks’ [reverse coded], ‘I am going to master the goal-scoring task because I can rely on my skills.’; (1) ‘does not apply’; (2) ‘does rather not apply’; (3) ‘rather does apply’; (4) ‘does apply’; Cronbach’s *α* = 0.824).

After completion of the task, the participants answered some additional questions – each on a 4-point-scale ranging from 1 to 4. The first of them – ‘During the goal-scoring task I thought about the cliché “Women are worse football players than men”’ – served as manipulation check (plus potential mediator). The second one – ‘The cliché “Women are worse football players than men” annoys me.’ – is an indicator of subjective vulnerability for sexist discrimination. The third and fourth items measured collective identification with women, respectively, female football players whilst completion of the task: ‘Whilst executing the goal scoring task I identified with the group of (women/female football players)’. Three more items were included in order to capture cognitive interference during task completion: ‘I thought about other things during the goal-scoring task.’, ‘Whilst completing the goal-scoring task I had difficulties to concentrate.’, and ‘My attention was completely focused on the goal-scoring task’ (reverse coded); Cronbach’s *α* = 0.741. Finally, the participants assessed the subjective difficulty of the goal-scoring task: ‘I perceived the goal scoring task as difficult’, with higher values indicating larger difficulty.

### Sample

Eighty^3^ female football players took part in the study. They were randomly assigned to the threat (*N* = 41) or no-threat (*N* = 39) condition. Their age ranged from 16 to 53, with a mean of 23.59 years (SD = 7.634). Their mean footballing experience was 12.09 years (SD = 6.042), ranging from 0 to 28 years. No participant quit during data collection or withdrew her consent afterwards. No cases were excluded from analysis.

## Results

Neither the items capturing importance, nor those referring to task-related self-efficacy – all measured before the experimental manipulation – showed a statistically significant difference between threat and no-threat condition; |*t*s| < 0.982, *p*s > 0.329 (concerning means and standard deviations of the scales or single-item measures mentioned above and below, see Table 1).

**Table 1:** Means and standard deviations.

	Overall	No threat	Threat
Variables	*M* (SD)	*M* (SD)	*M* (SD)
Importance	3.56 (0.509)	3.58 (0.466)	3.54 (0.552)
Task-related self-efficacy	2.91 (0.510)	2.90 (0.505)	2.91 (0.521)
Manipulation check	1.45 (0.967)	1.05 (0.320)	1.83 (1.202)
Subjective vulnerability for sexist discrimination	2.58 (1.134)	2.51 (1.211)	2.63 (1.067)
Collective identification with women	2.25 (1.131)	2.15 (1.159)	2.34 (1.109)
Collective identification with female football players	2.94 (0.985)	3.15 (0.933)	2.73 (1.001)
Cognitive interference	2.02 (0.750)	1.93 (0.742)	2.10 (0.757)
Subjective difficulty	2.41 (0.837)	2.38 (0.877)	2.44 (0.808)
Hits	1.71 (1.295)	2.00 (1.556)	1.44 (0.923)
Mistakes	0.71 (1.150)	0.64 (1.181)	0.78 (1.129)

The same applies to some of the items measured after task completion – namely those measuring cognitive interference (|*t*s| < 1.05, *p*s > 0.298), subjective difficulty of the goal-scoring task [*t*(78) = −0.289, *p* = 0.773], or identification with women [*t*(78) = −0.740, *p* = 0.462]. The participants within the threat condition (*M* = 2.73, SD = 1.001) did, however, identify somewhat less with female football players than those not threatened (*M* = 3.15, SD = 0.933): *t*(78) = 1.949, *p* = 0.055. Participants’ identification as female football players (*M* = 2.94, SD = 0.985) was stronger than that as women (*M* = 2.25, SD = 1.131), *t*(79) = −5.488, *p* < 0.001.

Whereas the subjective vulnerability for sexist discrimination did not significantly differ between conditions (*M*_threat_ = 2.63, SD_threat_ = 1.067; *M*_no threat_ = 2.51, SD_no threat_ = 1.211; *t*(78) = −0.476, *p* = 0.635), the manipulation check indicates that the intended stereotype threat worked: *M*_threat_ = 1.83, SD_threat_ = 1.202; *M*_no threat_ = 1.05, SD_no threat_ = 0.320; *t*(78) = −3.911, *p* < 0.001. It should be noted, however, that the majority of threatened participants (26, i.e. 63.4% versus 38, i.e. 97.4% of the non-threatened) chose the answer option ‘does not apply’.

In accordance with our hypothesis, threatened participants scored less hits (*M* = 1.44, SD = 0.923) than those not threatened (*M* = 2.00, SD = 1.556). In order to statistically test^4^ this difference, we conducted a multiple regression analysis including two control variables, namely years of footballing experience and task-related self-efficacy. The results, shown in Table 2, indicate the significant role of stereotype threat on shooting precision^5^.

**Table 2:** Regression analysis with scored hits as criterion.

Predictor and control variables	*B*	SE *B*	*β*	*t*	*p**^a^*	Uniquely explained variance (%)
				*t*(76)		
Stereotype threat (IV)^b^	−0.578	0.273	−0.224	−2.114	0.038	5.03
Task-related self-efficacy	0.754	0.276	0.297	2.728	0.008	8.38
Footballing experience	0.010	0.023	0.047	0.436	0.664	0.21

*R*^2^ = 0.144; $R_{{}_{adj}}^{{}^{{}^{2}}}=0.110$; *F*(3, 76) = 4.269, *p* = 0.008; VIFs < 1.06.

a *Two-tailed*.

b *No threat = 0; threat = 1*.

The number of mistakes, however (*M*_threat_ = 0.78, SD_threat_ = 1.129; *M*_no threat_ = 0.64, SD_no threat_ = 1.181) did not significantly differ. Again, a multiple regression analysis, with the same control variables, was conducted. The results are shown in Table 3.

**Table 3:** Regression analysis with mistakes as criterion.

Predictor and control variables	*B*	SE *B*	*β*	*t*	*p**^a^*	Uniquely explained variance (%)
				*t*(76)		
Stereotype threat (IV)^b^	0.154	0.249	0.067	0.617	0.539	0.45
Task-related self-efficacy	−0.663	0.252	−0.294	−2.631	0.010	8.22
Footballing experience	−0.008	0.021	−0.040	−0.360	0.720	0.15

*R*^2^ = 0.097; $R_{{}_{adj}}^{{}^{2}}=0.062$; *F*(3, 76) = 2.726, *p* = 0.050; VIFs < 1.06.

a *Two-tailed*.

b *No threat = 0; threat = 1*.

Concerning hits as well as mistakes, the role of task-related self-efficacy is almost identical – it explains 8.38% of the variance in the former and 8.22% in the latter. Footballing experience did not significantly contribute to variance explanation in either analysis. Thus, the pattern between the prediction of hits and mistakes deviates only for stereotype threat.

**Figure 2:**
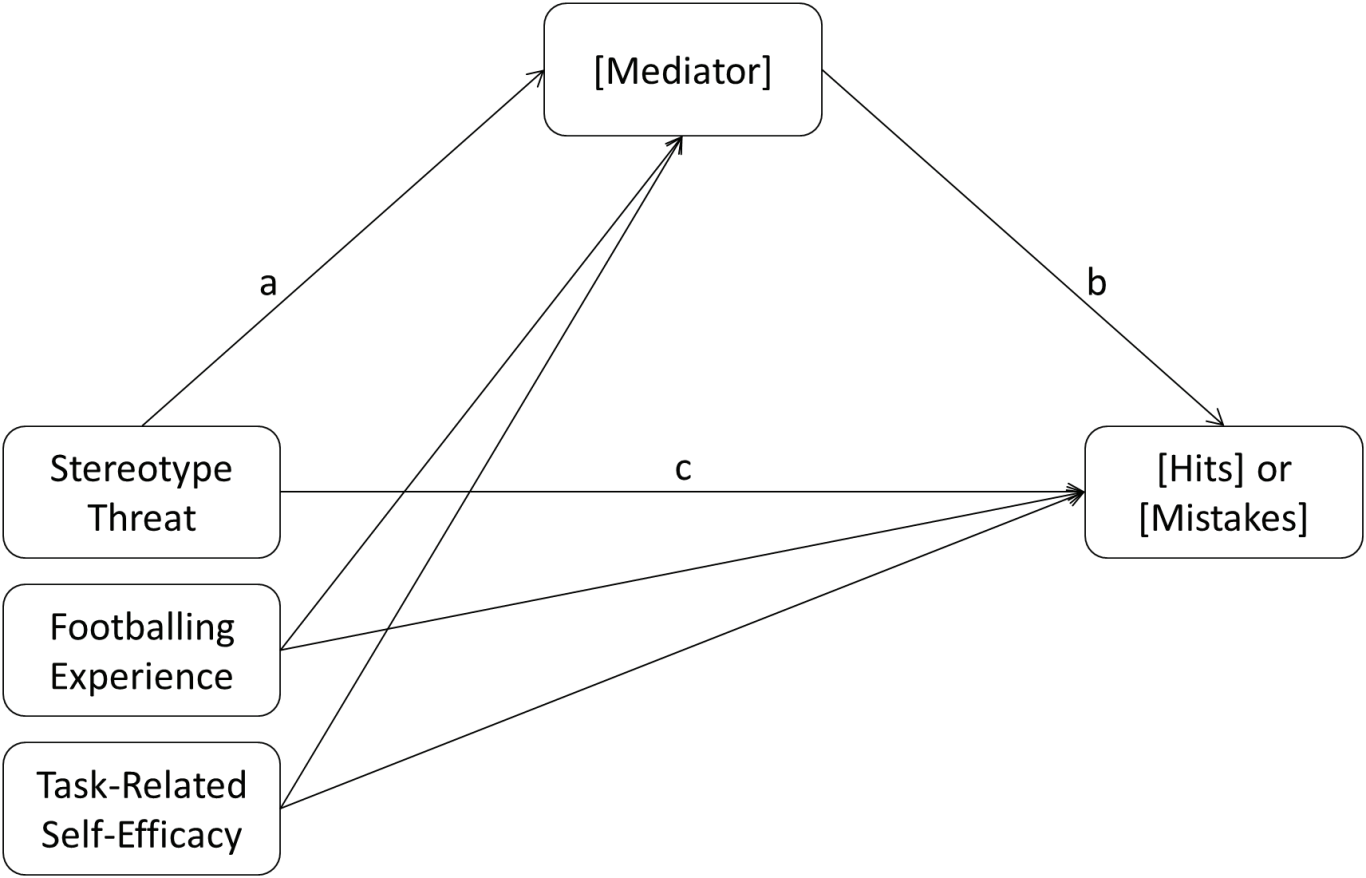
Mediation model.

In order to test potential indirect effects of stereotype threat on hits and mistakes, we conducted mediation analyses (see Figure 2 for a depiction of the model) as proposed by Hayes (2013).

The first six analyses contained stereotype threat as predictor, hits as criterion, task-related self-efficacy and experience as controls and one of the following potential mediators per calculation: thinking about the negative stereotype (our manipulation check), subjective vulnerability for sexist discrimination, collective identification with women, collective identification with female football players, cognitive interference, or subjective difficulty of the task. Each of the 95% confidence intervals for the indirect effect contained the 0 (10,000 bootstrap samples), indicating no statistically significant mediation effect. The second set of six analyses differed only concerning the criterion – now mistakes. Again, no statistically significant indirect effect was found.

Path a of the above mentioned analyses – between predictor and mediator – reached respectively approached statistical significance for two predictors: stereotype threat significantly affected thinking about the negative stereotype (*b* = 0.784, *β* = 0.408, *p* < 0.001) and collective identification with female football players (*b* = −0.418, *β* = −0.214, *p* = 0.055).

Additionally, we tested whether stereotype threat exerted an indirect effect on hits *via* mistakes. This could be another indicator of (conscious) cognitive interference as a mediator. Again, task-related self-efficacy and experience were included as control variables, and again, the 95% confidence interval included 0.

Before discussing implications of the study’s results, it should be considered whether a stereotype effect was – according to Keller’s (2008) criteria – likely to emerge. Apparently, the stereotype of women being unable to play football proper does fit excellently into the (experimental) situation and is directly applicable to the task. Results indicate that football (the relevant domain) is important to the participants: means for the respective items (≥3.45) are close the scale’s maximum (4). The demanding character of the task is indicated by different measures: the relatively low number of hits (overall mean = 1.71), the overall mean (2.41, i.e. above the scale midpoint) for the subjective difficulty measure, and some participants’ oral feedback after test sessions. The impression of one’s performance being under scrutiny is highly likely to have arisen amongst participants due to the test setting and procedure. Again, participant feedback confirms this assumption. Summing up, it appears reasonable to consider the four criteria met and expect a stereotype effect likely to emerge.

## Discussion

In line with our hypothesis, threatened participants scored less hits. They did not, however, make significantly more mistakes during task execution. Thus, the rather difficult motor task – tapping in well-learned, automated motion sequences – was impeded when the negative stereotype was salient; the mainly cognitive task of following instructions correctly, was not. This is surprising insofar as the stereotype threat effect was introduced into the psychological literature as one affecting cognitive/intellectual tasks (Steele and Aronson, 1995). The same applies to our finding that neither one of the three single indicators of cognitive interference nor the respective scale were affected by threat. Still, it is not impossible that the stereotype threat affected working memory *via* verbal ruminations or worries (Rydell et al., 2009); both concepts are not (well) captured by our measures of cognitive interference. In this case, however, one would expect both fewer hits and more mistakes within the threat condition. It should be noted that the average number of mistakes is rather low (0.71 overall), which might be indicating a comparably low difficulty of the task of following the instructions correctly – making the manifestation of a stereotype effect on this particular measure less likely. Employing a more sensitive measure of mistakes and/or more demanding instructions seem appropriate strategies for potential replications of our study.

The fact that stereotype threat hampered goal scoring complies with the reasoning of Schmader et al. (2008) insofar as their integrated process model predicts an effect on sensorimotor tasks. Indeed, it seems plausible that the threat ‘in combination with the motivation to disconfirm the stereotype, translates into a strong motivation to avoid failure’ which in turn lets the participants ‘focus attention on themselves and their performance, becoming more vigilant to detect signs of failure’ (Schmader et al., 2008, p. 343). Keller and Dauenheimer (2003) hypothesised a mediation of stereotype threat effects on performance *via* regulatory focus and tested it with specific emotions typical of one or the other regulatory focus; they found empirical support for at least one kind of emotions: dejection significantly mediated the effect of gender-related stereotype threat on women’s performance in a mathematics test. Seibt and Förster (2004) argue similarly when they introduce regulatory focus as mediator between stereotype threat and performance: being confronted with a negative stereotype would induce a prevention focus, whereas positive stereotypes foster a promotion focus. The former is associated with ‘safety and nonlosses’, the latter with ‘advancement and gains’ (Seibt and Förster, 2004, p. 39). Applied to our study, with accuracy at the core of the task, however, both regulatory foci should motivate the participants to score as many hits as possible, either in order to maximise a positive outcome (i.e., hits) or to minimise a negative outcome (i.e., misses). Concerning this measure, thus, our results do not support this line of reasoning. The same applies to the number of mistakes: ‘promotion focus emphasis on strategic eagerness should lead to a riskier processing style’ (Seibt and Förster, 2004, p. 39) presumably resulting in more mistakes, ‘whereas prevention focus emphasis on strategic vigilance should lead to a more careful processing style’ (Seibt and Förster, 2004, p. 40), presumably resulting in fewer mistakes. The number of mistakes, however, did not differ significantly. Because we did not assess the participants’ regulatory focus, an evaluation of our results from this particular perspective lacks empirical data. Therefore, the above reasoning needs to be considered cautiously. With regard to Schmader et al. (2008), our measures of cognitive interference could be interpreted as indicators of ongoing monitoring processes. In this case, threatened participants should score significantly higher on the respective items, which is not the case. Nevertheless, we consider it plausible that the pursuit of a certain regulatory focus could be a strategy in order to maintain high self-esteem when an individual’s collective identity is threatened. Further research, integrating social identity and regulatory focus approaches, could help to understand the respective constructs’ relations better.

Moreover, the lack of influence of threat on (our indicators for) cognitive performance goes hand in hand with a lack of evidence for an assumed mediating effect on motor tasks: our findings do not corroborate the idea that stereotype threat strains sparse cognitive resources. An explanation considering threat’s impact on collective identification appears more promising. Admittedly, the indirect path of threat *via* identification as female football player on hits was not statistically significant. However, at least the path from threat to identification approached statistical significance (*p* = 0.055). An interesting aspect of this finding is the contrast to the second measure of collective identification – that with women *per se* – where threat made no difference. According to the SCT, ‘given two equally “accessible” categories, the one that better “fits” the perceptual data will become salient’ (Turner et al., 1987, p. 55). Here, in the context of a football-related task, the narrower, more specific social group of female football players can reasonably be expected to fit better than the broader, rather unspecific group of females *per se* (both categories can be expected to be very well accessible to our participants). The overall means of identification reflect this assumption as the participants identified more strongly as female football players than as women. In the light of the SCT, the negative impact of our manipulation on the identification as female football players, but not females *per se*, makes perfect sense: apparently, they employed an individual mobility strategy in order to maintain self-esteem. Because identification was measured after the goal scoring task, it is also feasible that poor performance mediated the effect. However, this was not found to be the case.

Although we did not find compelling evidence for an influence of identification as female football player on the task, such a relationship between collective identification and performance should not be categorically ruled out. In fact, the respective link is well established, e.g. in the context of organisational outcomes (Haslam et al., 2003), or *via* motivation (Van Knippenberg, 2000); moreover, social identity is perceived as major factor in leadership – hence, again related to performance (Haslam et al., 2011). Further research should employ different operationalisations of a wider range of collective identities and consider potential moderators or mediators. Moreover, future samples should be larger in order to secure high statistical power. Although we found a significant effect of threat on hits, a lack of power may have contributed to our not being able to detect a full mediation, or a more than only marginally significant effect of threat on identification as female football player.

Another fruitful idea for future research could be an investigation into task-related self-efficacy as a potential mediator. Given its reliable and comparably strong influence on hits as well as mistakes, it appears worthwhile to test whether a stereotype threat reduces task-related self-efficacy.

Summing up, we could demonstrate the stereotype effect for a complex and demanding motor task. In the light of the ongoing replicability crisis (e.g. Świątkowski and Dompnier, 2017), our findings can therefore strengthen the confidence in the existence of the stereotype effect. Hypothesised mediational paths could, however, not be corroborated. Especially interesting is the absence of evidence for an influence of threat *via* cognition on motor performance. Therefore, it might be wise to take a step back and reconsider possible pathways between stereotype threat and motor tasks, then try to replicate the study and bolster it with measures relating to this re-developed theory.

## Ethics Statement

This study was carried out in accordance with the ‘Berufsethische Richtlinien des Berufsverbandes Deutscher Psychologinnen und Psychologen e.V. und der Deutschen Gesellschaft für Psychologie e.V.’ (professional ethics guidelines of the occupational union of German psychologists and the German society of psychology). All subjects gave written informed consent in accordance with the Declaration of Helsinki. An ethics approval was not required as per the university’s guidelines or national regulations. The research presented no discernible risk to the participants.

## Author Contributions

HG wrote the article and supervised data collection. MK contributed the initial research idea, collected the data, and contributed information during writing. HG and MK developed the research idea and hypotheses, designed the study, and analysed the data.

### Conflict of Interest Statement

The authors declare that the research was conducted in the absence of any commercial or financial relationships that could be construed as a potential conflict of interest.

## Footnotes

^1^Following John Cleese’s convincing argumentation (Vaske, 2006), we use the English term football for the sport that is in some areas of the world also known as associate football, or soccer.

^2^Differences between the conditions are italicised here. Of course, during the study, the respective texts were presented without highlighting.

^3^An *a priori* power analysis employing G*Power 3.1.9.2 (Faul et al., 2009) yielded a target sample size of 74, given an effect size *f^2^* = 0.15 and power = 0.95 for a one-tailed, or of 89 for a two-tailed test. *Post hoc*, calculated with the actual sample size of *N* = 80, and an effect size of *f^2^* = 0.058 [calculated as proposed by Selya et al. (2012)], the power equals 0.691 (one-tailed), or 0.569 (two-tailed). Data collection stopped when the sample size was considered sufficiently large – before any analyses were conducted.

^4^Due to conventions, we conducted a two-tailed test in spite of our directional hypothesis.

^5^Without consideration of the control variables, the effect of stereotype threat on hits reaches a level of significance of *p* = 0.052 (*t*(78) = 1.973), indicating a suppressor effect.
